# A multi-centre cohort study shows no association between experienced violence and labour dystocia in nulliparous women at term

**DOI:** 10.1186/1471-2393-11-14

**Published:** 2011-02-21

**Authors:** Hafrún Finnbogadóttir, Elisabeth Dejin-Karlsson, Anna-Karin Dykes

**Affiliations:** 1 Department of Nursing, Faculty of Health and Society, Malmö University, Malmö, Sweden; 2 Division of Nursing, Department of Health Sciences, Medical Faculty, Lund University, Lund, Sweden

## Abstract

**Background:**

Although both labour dystocia and domestic violence during pregnancy are associated with adverse maternal and fetal outcome, evidence in support of a possible association between experiences of domestic violence and labour dystocia is sparse. The **aim **of this study was to investigate whether self-reported history of violence or experienced violence during pregnancy is associated with increased risk of labour dystocia in nulliparous women at term.

**Methods:**

A population-based multi-centre cohort study. A self-administrated questionnaire collected at 37 weeks of gestation from nine obstetric departments in Denmark. The total cohort comprised 2652 nulliparous women, among whom 985 (37.1%) met the protocol criteria for dystocia.

**Results:**

Among the total cohort, 940 (35.4%) women reported experience of violence, and among these, 66 (2.5%) women reported exposure to violence during their first pregnancy. Further, 39.5% (n = 26) of those had never been exposed to violence before. Univariate logistic regression analysis showed no association between history of violence or experienced violence during pregnancy and labour dystocia at term, crude OR 0.91, 95% CI (0.77-1.08), OR 0.90, 95% CI (0.54-1.50), respectively. However, violence exposed women consuming alcoholic beverages during late pregnancy had increased odds of labour dystocia, crude OR 1.45, 95% CI (1.07-1.96).

**Conclusions:**

Our findings indicate that nulliparous women who have a history of violence or experienced violence during pregnancy do not appear to have a higher risk of labour dystocia at term, according to the definition of labour dystocia in this study. Additional research on this topic would be beneficial, including further evaluation of the criteria for labour dystocia.

## Background

Accumulating knowledge suggests that domestic violence occurring during pregnancy is a serious public health issue due to the risk for adverse maternal and fetal health outcomes [1-3]. Labour dystocia, another serious complication in obstetrics, has also been increasingly highlighted during the past decades [4-9]. Labour dystocia is defined as a slow or difficult labour or childbirth. According to Kjaergaard et al. [10] the term 'dystocia' is frequently used in clinical practice, yet there is no consistency in the use of terminology for prolonged labour or labour dystocia [4,6,11,12]. However, labour dystocia accounts for most interventions during labour [4,6,7]. Although both labour dystocia [4,7] and domestic violence during pregnancy [1,2] are associated with adverse maternal and fetal outcome, evidence in support of a possible association between experiences of violence and labour dystocia is sparse. One recent study from Iran has shown an association between experienced abuse by an intimate partner and labour dystocia, and such abuse included psychological threats as well as physical, or sexual abuse [13].

Although the demographic background of women exposed to domestic violence may vary widely, some women are more vulnerable and at increased risk [14]. Disadvantaged women, with low socio-economic status [15-17] and younger age, [18] as well as single women at younger age, [15-17] certain ethnic groups [15,17,19] and even women with a partner born outside Europe [17] are more likely to be exposed to domestic violence. Also unhealthy maternal behaviour such as smoking [20-23] and use of alcohol and drugs during pregnancy are more common among women who live in violent relationships [20,21]. Pregnant women exposed to violence have a greater risk of delivering babies with low birth weight, [20,22,24] premature labour, [22,25] abruption of placenta [25] and fetal trauma [22,24,25] or death [22,24,26] and are also at increased risk of caesarean section [25].

Some identified risk factors for dystocia are high maternal age, [10,11] short maternal height, [27,28] overweight, [10] obesity [29] and smoking [30]. Also, high fetal weight increases the risk for prolonged labour [31] and labour dystocia [32]. Further, up to 50% of unplanned caesarean sections among nulliparous women are related to labour dystocia [4,6].

Already thirty years ago, Lederman et al. [33] showed that physical and psychosocial characteristics of the woman, such as maternal emotional stress related to pregnancy and motherhood, partner and family relationships, and fears of labour were significantly associated with less efficient uterine function, higher state of anxiety, higher epinephrine levels in plasma and longer length of labour. The higher levels of epinephrine may disrupt the normal progress in labour or the coordinated uterine contractions explained by an adrenoreceptor theory [34]. Subsequently, Alehagen et al. [35] confirmed significantly increased levels of all three stress hormones from pregnancy to labour and drastically increased levels of epinephrine and cortisol compared with nor-epinephrine, indicating that mental stress is more dominant than physical stress during labour. Maternal psychosocial stress, family functioning and fear of childbirth may have an association with specific complications such as prolonged labour or caesarean section [36]. History of sexual violence in adult life is associated with an increased risk of extreme fear during labour, [37] and fear of childbirth in the third trimester has been shown to increase the risk of prolonged labour and emergency caesarean section [38]. Thus, the current body of evidence in this area would support the hypothesis that experience of violence before and/or during pregnancy increases the risk of labour dystocia.

The aim of this study was to investigate whether self-reported history of violence or experienced violence during pregnancy is associated with increased risk of labour dystocia in nulliparous women at term.

## Methods

The material used in this study originates from the Danish Dystocia Study (DDS), a population-based multi-centre cohort study, and 8099 nulliparous women were potentially eligible for inclusion in the study [8-10]. However, 6356 women were invited to the DDS study (external drop-out was 21.5%) and 5484 women accepted participation. For the current sub-study, a data set on 2652 nulliparous women who fulfilled the inclusion criteria (showed below) was available for analyses of exposure to violence before and during pregnancy. Among these, 985 (37.1%) met the protocol criteria for labour dystocia (Table 1). These diagnostic criteria are in accordance with the American College of Obstetrics and Gynecology (ACOG) criteria for dystocia in labour's second stage [6] and also with the criteria for labour dystocia in first and second stage described by the Danish Society for Obstetrics and Gynecology [39,40]. The diagnosis prompted augmentation (i.e. with oxytocin stimulation) [8-10].

**Table 1:** Definition of stages and phases of labour and diagnostic criteria for dystocia for current sub-study [8-10].

Stage of labour	Definition of stages and phases	Diagnostic criteria for dystocia
***First stage***	***From onset of regular contractions leading to cervical dilatation***	

Latent phase	Cervix dilatation 0 - 3.9 cm	Not given in this phase

Active phase	Cervix dilatation ≥ 4 cm	< 2 cm assessed over four hours

***Second stage***	***From full dilatation of cervix until the baby is borne***	

Descending phase	From full dilatation of cervix to strong and irresistible urge to push	No descending ≥ 2 hours or ≥ 3 hours if epidural was administrated

Expulsive phase	Strong and irresistible pushing during the major part of the contractions	No progress ≥ 1 hour

Data were collected prospectively between May 2004 and July 2005. Participants were recruited from nine obstetric departments in Denmark with annual birth rates between 850-5400 per year. The departments were four large university hospitals, three county hospitals, and two local district departments. Recruitment of the women took place in the antenatal clinics at 33 gestational weeks, and baseline information was collected at 37 gestational weeks. *Inclusion criteria *were Danish speaking (i.e. reading/understanding) nulliparous women at 18 years of age or older, with a singleton pregnancy in cephalic presentation and no planned elective caesarean section or induction of labour. *Exclusion criteria *were nulliparous women with a delivery < 37 or > 42 weeks of gestation, induction, elective caesarean section and breech presentation (n = 1115 or 17.5% in DDS). All data were based on a self-administrated questionnaire and on information contained in obstetric records filled out by the midwives at admission and postpartum. Forty percent of the questionnaires were completed in an internet version. Fourteen (0.5%) of the 2652 women did not answer the questions about violence and were classified as having no exposure to violence.

Eight items in the questionnaire dealt with violence and originated from the short form of the Conflict Tactics Scale (CTS2S) [41]. This instrument has been used in large population-based studies in Denmark, and translation from English to Danish and back translation to English were performed prior to the Danish Health and Morbidity survey 2000 [42]. The questions were adapted for a pregnant cohort in the DDS [8-10]. Three alternatives were provided as possible answers to the various exposure questions: 'yes during this pregnancy', 'yes earlier', and 'no never'. Women were not required to provide information concerning the number of episodes of violence that had occurred (Additional file 1).

'*History of violence' *was defined as experience of violence ever in lifetime before and/or during pregnancy, *'Violence before pregnancy' *as experienced violence ever in lifetime before pregnancy, '*Violence during pregnancy' *as experienced violence during pregnancy (with or without violence before pregnancy) and '*Violence for the first time during pregnancy' *as experienced violence during pregnancy without experienced violence before pregnancy.

Further, for the purpose of analysis, violence was categorized as i) threat of violence, ii) physical violence, iii) sexual violence, and iv) serious violence. However, a more detailed description of the prevalence of violence will be published elsewhere by another research group.

For the purpose of the current sub-study, the concept *domestic violence *was defined as exposure to psychological and/or physical abuse by 'Your husband/Co-habitant' or 'A person you know very well in your family', according to the first two alternatives in question 9 in the questionnaire (Additional file 1).

Background and lifestyle factors were classified as follows. *Maternal age *was classified as 18-24, 25-29, 30-34 and >34 years. *Country of origin *was classified according to whether the woman was born in Denmark, in another Nordic country, or in other country. *Cohabiting status *was divided into yes or no. *Educational status *was dichotomised as ≤ 10 years or > 10 years and *employment status *as employed or unemployed (including voluntary unemployed or studying). *Smoking status *was classified as "yes" (if the woman was a daily smoker or was smoking at some point during pregnancy) or "no" (never smoked or alternatively, if she had ceased before pregnancy) and *use of alcohol *as "yes" (if the woman had been drinking alcohol during pregnancy at the time when the questionnaire was administered) or "no" (if the woman had been drinking solely alcohol-free drinks). *Body mass index *(BMI) was calculated from maternal weight and height before the pregnancy and classified as normal or low weight if BMI was ≤ 25, or overweight when > 25. Infant *birth weight *was dichotomised as < 3500 g or ≥ 3500 g and *delivery mode *as partus normalis (PN) or instrumental delivery, including caesarean section and vacuum extraction (VE).

### Ethics

Since no invasive procedures were applied in the study, no Ethics Committee System approval was required by Danish law. The policy of the Helsinki Declaration was followed throughout the data collection and analyses. Written consent was obtained and person-specific data were protected by codes. Permission to establish the database was obtained from the Danish Data Protection Agency (j. no. 2004-41-3995).

### Statistical methods

Chi-square analysis was used to investigate differences in background characteristics between women who were exposed to violence and women not exposed to violence. Odds ratios (OR) and 95% confidence intervals (95% CI) were calculated for the crude associations between various background- and lifestyle characteristics and labour dystocia, with dystocia as the dependent variable for logistic regression. Age was dichotomised as ≤ 24 or >24 years and country of origin as Danish or non-Danish. Univariate logistic regression was used to analyse the crude odds ratios for dystocia in relation to various background- and lifestyle characteristics and self-reported history of violence. Further, multiple regression was used to analyse domestic violence (solely) and history of violence as independent variables (two different analysis) together with the other well-documented maternal factors (maternal age, BMI and smoking) associated with dystocia. Odds ratios were used as estimates of relative risk. Statistical significance was accepted at p < 0.05. Statistical analyses were performed using the Statistical Package for Social Sciences (SPSS) version 16.0 for Windows.

## Results

Table 2 provides a descriptive overview of the maternal characteristics for the total cohort of 2652 women, with and without self-reported experience of 'history of violence', 'violence before pregnancy' and 'violence during pregnancy'.

**Table 2:** Descriptive overview of maternal characteristics in nulliparous women who have reported experienced violence **before and/or during pregnancy **compared to women not exposed to violence (n = 2652).

**Characteristics**	**Total**	**History of violence**		**P** **(2-sided)**	**Violence** **before pregnancy**		**P** **(2-sided)**	**Violence** **during pregnancy**		**P** **(2-sided)**
					
	**n (%)**	**Not exposed** **n (%)**	**Exposed** **n (%)**		**Not exposed** **n (%)**	**Exposed** **n (%)**		**Not exposed** **n (%)**	**Exposed** **n (%)**	
**Total**	2652 (100.0)	1712 (64.7)	940 (35.4)		1738 (65.5)	914 (34.5)		2586 (97.5)	66 (2.5)	
**Age, years**										
18 - 24	440 (16.5)	233 (13.6)	207 (22.1)	< 0.001	236 (13.6)	204 (22.4)	< 0.001	420 (16.3)	20 (30.8)	0.02
25 - 29	1300 (49.0)	884 (51.6)	416 (44.4)		901 (51.8)	399 (43.8)		1274 (49.3)	26 (40.0)	
30 - 34	728 (27.5)	476 (27.8)	252 (26.9)		481 (27.7)	247 (27.1)		712 (27.6)	16 (24.6)	
> 34	180 (6.8)	119 (7.0)	61 (6.5)		120 (6.9)	60 (6.6)		177 (6.7)	3 (4.6)	
*Missing*	4 (0.2)									
**Country of** **origin**										
Denmark	2452 (92.5)	1577 (92.1)	875 (93.1)	NS	1603 (92.2)	849 (92.9)	NS	2390 (92.4)	62 (93.9)	NS
Nordic countries	54 (2.0)	38 (2.2)	16 (1.7)		38 (2.2)	16 (1.8)		53 (2.0)	1 (1.5)	
Other countries	146 (5.5)	97 (5.7)	49 (5.2)		97 (5.6)	49 (5.4)		143 (5.5)	3 (4.5)	
*Missing*	0 (0.0)									
**Cohabiting** **status**										
Yes	2517 (94.9)	1645 (99.7)	872 (98.8)	0.004	1668 (99.7)	849 (98.7)	0.003	2461 (99.5)	56 (94.9)	< 0.001
No	16 (0.6)	5 (0.3)	11 (1.2)		5 (0.3)	11 (1.3)		13 (0.5)	3 (5.1)	
*Missing*	119 (4.5)									
**Education** **status**										
> 10 years	2128 (80.3)	1436 (84.7)	692 (74.7)	< 0.001	1457 (84.6)	671 (74.6)	< 0.001	2083 (81.5)	45 (68.2)	< 0.006
≤10 years	494 (18.6)	260 (15.3)	234 (25.3)		265 (15.4)	229 (25.4)		473 (18.5)	21 (31.8)	
*Missing*	30 (1.1)									
**Employment** **status**										
Employed	1849 (69.7)	1237 (74.1)	612 (66.8)	< 0.001	1255 (74.0)	594 (66.7)	< 0.001	1805 (71.6)	44 (68.8)	NS
Unemployed	737 (27.8)	433 (25.9)	304 (33.2)		441 (26.0)	296 (33.3)		717 (28.4)	20 (31.2)	
*Missing*	66 (2.5)									
**Smoking**										
No	1995 (75.2)	1377 (80.9)	618 (65.9)	< 0.001	1396 (80.8)	599 (65.7)	< 0.001	1953 (75.9)	42 (63.6)	0.022
Yes	645 (24.3)	325 (19.1)	320 (34.1)		332 (19.2)	313 (34.3)		621 (24.1)	24 (36.4)	
*Missing*	12 (0.5)									
**Use of alcohol**										
No	1895 (71.5)	1240 (77.0)	655 (73.3)	NS	1256 (76.7)	639 (73.6)	NS	1851 (75.0)	44 (69.8)	NS
Yes	637 (24.0)	398 (24.3)	239 (26.7)		408(24.5)	229 (26.4)		618 (25.0)	19 (30.2)	
*Missing*	120 (4.5)									
**BMI**										
Normal or low (≤ 25)	1954 (73.7)	1261 (77.5)	693 (77.9)	NS	1282 (77.6)	672 (77.8)	NS	1902 (77.6)	52 (80.0)	NS
Overweight (> 25)	563 (21.2)	366 (22.5)	197 (22.1)		371 (22.4)	192 (22.2)		550 (22.4)	13 (20.0)	
*Missing*	135 (5.1)									

Statistical significance is accepted at p < 0.05

^† ^Same women can occur in more than one group

Among the 940 (35.4%) women who reported experience of 'history of violence', 914 (97.2%) reported experienced 'violence before pregnancy'. Also, 66 (2.5%) women reported violence during current pregnancy (Table 2). Of these women, 26 (39.5%) were exposed to 'violence for the first time during pregnancy'. All women exposed to violence for the first time during their first pregnancy were Danish, three (11.5%) women in the age group 18 - 24 years, 17 (65.4%) at age 25- 29, five (19.2%) at age 30-34 and one (3.8%) >34 years. Three (11.5%) women were not cohabiting, five (19.2%) had ≤ 10 years education, eight (30.8%) were unemployed, seven women were smokers (26.9%), ten (38.4%) were alcohol consumers at the 37^th ^week of gestation, and five (19.2) had BMI > 25.

Of the 940 women who had a 'history of violence', 697 (77%) answered a question concerning whom the perpetrator was. Thirty-seven percent had been exposed to domestic violence. Further, 22% to violence by someone they knew very well (not family member) and 15% by someone they knew superficially (family or other). The perpetrator was a stranger in 26% of the cases. Of the 66 women who had been exposed to violence during pregnancy, 53 (80%) answered the question about the perpetrator, and in 23 (43.0%) cases they were exposed to domestic violence.

The median age of all nulliparous women was 28 years. In the violence-exposed group significantly more women were in the 18-24 age categories in all three violence exposure groups (p < 0.001, p < 0.001, p = 0.020). No differences in exposure to violence were found in relation to country of origin. In the total sample, 94.9% of the women (n = 2517) were cohabiting. Across all categories of exposure to violence, such exposure was proportionally more often reported by non-cohabiting women (p = 0.004, p = 0.003 respectively p < 0.001) albeit only 16 (0.6%) of the women were not cohabiting. Slightly more than eighty percent (80.3%) of the women had more than 10 years of schooling. Exposure to 'history of violence' and 'violence before pregnancy' was more frequently reported by women who had a lower educational level (≤ 10 years) compared to women not exposed (p < 0.001), as well as in the group 'violence during pregnancy' (p < 0.006). Over two-thirds (69.7%) of the women were employed. The exposed group differed from the non-exposed group before pregnancy in that more women were unemployed (p < 0.001). However, there was no significant difference in employment status among the group of 66 (2.5%) women who were violence-exposed during pregnancy (Table 2).

More than twenty-four percent (24.3%) of these nulliparous women were smokers at term or at some point during pregnancy. Exposure to violence was proportionally more often reported by smokers than by non-smokers across all categories (p < 0.001, p < 0.001, p = 0.022). Twenty-four percent of the nulliparous reported that they consumed alcohol during pregnancy, in 37^th ^week of pregnancy (Table 2). The quantity ranged between 1 to 10 units of alcoholic beverages per week. However, there were no significant differences in alcohol consumption between violence-exposed or unexposed women. No differences in exposure to violence were found in relation to BMI.

Crude odds ratios showed no association between experiences of 'history of violence' and dystocia (n = 940) OR 0.91, 95% CI (0.77-1.08), 'violence before pregnancy' and dystocia (n = 914) OR 0.90, 95% CI (0.77-1.07), 'violence during pregnancy' and dystocia (n = 66) OR 0.90, 95% CI (0.54-1.50), or 'first time violence during pregnancy' (n = 26) OR 1.24, 95% CI (0.56-2.71) and dystocia. Moreover, no significant associations were found between dystocia at term and any of the various categorizations of violence: i) 'threat of violence' OR 0.97, 95%CI (0.79-1.18), ii) 'physical violence' OR 0.93, 95%CI (0.78-1.11), iii) 'sexual violence' OR 1.18, 95%CI (0.85-1.62) and iv) 'serious violence' OR 1.00, 95%CI (0.81-1.23).

A multiple regression done with 'domestic violence' (solely) as an independent variable together with already known factors as maternal age, BMI and smoking associated with dystocia showed no significant association to dystocia at term, OR 1.23 95% CI (0.89 - 1.69). Women older than 24 years and women with pre pregnancy overweight had significantly increased risk for dystocia at term with OR 1.53 95% CI (1.16 -2.00) respectively OR 1.31 95% CI (1.07-1.62). Further, multiple regression with 'history of violence' as an independent variable together with age, BMI and smoking showed no association to dystocia at term with OR 0.98 95% CI (0.81-1.18).

Table 3 shows the relationship between background and lifestyle characteristics and the risk (crude odds ratios) for dystocia in women with and without exposure to 'history of violence'. Women older than 24 years had significantly increased risk for dystocia at term, irrespective of exposure to violence (exposed: OR 1.64, 95% CI: 1.16-2.30; unexposed: OR 1.36, 95% CI: 1.02-1.83). Also, women who consumed alcohol during pregnancy and had experienced exposure to 'history of violence' had an increased risk for dystocia at term (exposed: OR 1.45, 95% CI: 1.07-1.96).

**Table 3:** Maternal background characteristics as risk factors for dystocia in nulliparous women with and without experience of history of violence, as shown by crude odds ratios (OR) and 95% confidence intervals.

**Characteristics**	**History of violence** **(n = 940)**		**No history of violence** **(n = 1712)**	
	
	**Total cases of dystocia** **(n = 337)**		**Total cases of dystocia** **(n = 648)**	
	**Dystocia/no dystocia**	**OR 95% CI**	**Dystocia/no dystocia**	**OR 95% CI**
Age > 24 years	279/449	1.64 (1.16-2.30)	574/905	1.36 (1.02-1.83)
Non-Danish	21/44	0.84 (0.49-1.44)	58/77	1.26 (0.88-1.80)
Not cohabiting	4/7	1.02 (0.29-3.52)	1/4	0.41 (0.05-3.64)
Low educational status (≤10 years)	85/149	1.00 (0.74-1.38)	84/176	0.76 (0.57-1.00)
Unemployed	120/183	1.23 (0.93-1.63)	154/279	0.89 (0.71-1.12)
Smoking	118/202	1.06 (0.80-1.41)	122/203	0.98 (0.77-1.26)
Alcohol consumption	100/139	1.45 (1.07-1.96)	144/254	0.93 (0.74-1.18)
Overweight > 25 BMI	80/117	1.26 (0.91-1.75)	156/210	1.26 (0.99-1.60)

Women giving birth to an infant with a birth weight of 3500 g or more (n = 1231) had significantly increased risk of dystocia irrespective of exposure to violence (exposed (n = 424): OR 2.0, 95% CI: 1.49-2.69; unexposed (n = 807): OR 1.39, 95% CI: 1.12-1.71). Women with dystocia had significantly increased risk for instrumental deliveries (n = 632) compared to normal deliveries, irrespective of exposure to violence (exposed (n = 221): OR 4.45, 95% CI: 3.23-6.11; unexposed (n = 410): OR 4.21, 95% CI: 3.33-5.33).

## Discussion

More than one third (35.4%) of the women in this study had been exposed to violence ever in their lifetime, i.e. before and/or during pregnancy. However, no association was found between experienced violence and labour dystocia in nulliparous women at term. Therefore, our findings suggest that women who have been exposed to violence ever in lifetime before and/or during pregnancy are not at a higher risk of prolonged delivery process at term. However, as this is the first study ever with the specific aim to examine the potential association between history of violence and labour dystocia, the current results should be regarded as only preliminary, and further research is needed in order to confirm these apparently negative findings. Nevertheless, recent findings by Khodakarami et al.[13] did show an association between experienced intimate partner violence and labour dystocia. However, Khodakarami et al.[13] did not define dystocia, and also, our definition of experienced domestic violence is somewhat broader, which makes it difficult to compare the results. Yet, in our study, the odds of having dystocia if exposed solely to domestic violence were increased by 23%, albeit not significantly. These two major challenges in obstetrics thus appear mostly to have different underlying risk factors, although smoking is common to both exposure to violence [20-23,30] and prolonged labour [30], which can in turn lead to labour dystocia.

The subjects investigated in our study are primarily Danish women (92.5%), i.e. they were born in Denmark and have Danish ethnicity. Due to ethical considerations, women younger than 18 years were excluded in this study in respect for Danish law regarding autonomy, because otherwise parental consent would have been necessary for participation in the study.

The mean age of the nulliparous women was rather high, i.e. 28 years. In accordance with results from previous studies, [16-18] younger age (< 24 years) is a risk group for exposure to violence. The results in our study showed that women older than 24 years with or without experience of violence had significantly increased risk for dystocia at term, although in the non-violence exposed group, the association may be regarded as marginally significant due to the lower limits of the confidence interval. Earlier studies have shown that increasing maternal age has a strong association with labour dystocia [10,11].

Women exposed to violence were more often smokers, in accordance with what several international studies have shown, [21-23] even though smoking has been decreasing in Denmark during the last decade, especially in the age-group 25-44 years [42]. A nation-wide study in Denmark showed that in the year 2005, smoking prevalence at some point in pregnancy was 16% [43]. However, our study had the same definition of smoking as in the study of Egebjerg Jensen et al.[43], and the prevalence of smoking during pregnancy was higher, i.e., 24.3% in our study. It is alarming if the smoking prevalence is increasing during pregnancy.

Another background variable that might be of importance for an association between exposure to violence and labour dystocia is alcohol. In the current study, women who had experience of violence and who also were alcohol consumers during late pregnancy had higher risk of dystocia at term compared to non-violence exposed women. The calculated odds ratio was significant (p = 0.017), albeit the strength of the association may perhaps best be regarded as modest in the current context, in that these are crude odds ratios, i.e. unadjusted for any other background characteristics. In accordance with earlier results, [20,21] unhealthy maternal behaviour such as use of alcohol and drugs during pregnancy are more common among women who live in violent relationships. Yet, to our knowledge associations between consumption of alcohol during the third trimester in pregnancy and experience of violence as a risk factor for labour dystocia have not been described in the literature before. These findings are difficult to interpret and need further investigation.

In the present study 2.5% (n = 66) of nulliparous women were exposed to violence during the pregnancy and 39.5% (n = 26) of them had never been exposed to violence previously. Thus, the violence was initiated during their first pregnancy. The size of this group was however limited and these results would need to be investigated further. Transition into a new social role can be experienced as a very stressful event for the father to-be [44] and may lead to increased pre-existing strains in the couple's relationship to such an extent that the partner uses psychological or physical violence towards the mother to-be. However, our definition of 'history of violence' in this study includes all experienced violence during and before pregnancy, and thus, intimate partner violence is only one possible component.

It should be noted that the current results regarding prevalence of exposure to violence may conceivably represent an underestimate of the true rates. Technical errors affected the internet data collection (40% of the material), such that women were unable to report whether they were exposed to violence during current pregnancy or not. More specifically, they were only provided with two alternatives of answers in the questionnaire, instead of three. Also, the true prevalence of physical and psychological abuse in pregnant women is difficult to estimate since women who are exposed to violence may be afraid to report such violence in fear of abuse escalation [24]. First time pregnancy may escalate existing stressors in the couple's relationship which can lead to psychological or physical abuse and this in turn may result in prolonged labour [33-36]. Nevertheless, in the current study, there was no association between exposure to 'first time violence during pregnancy and dystocia'. However, there were only 26 women in this group. Despite the limited size of this group, the odds of having dystocia were increased by almost 25%, albeit not significantly. Thus, the question remains as to whether a significant association between dystocia and exposure to first time violence during pregnancy would be obtained in a larger sample. A potential weakness in the current study is the small number of individuals in some of the sub-group analyses.

In current study overweight pre pregnancy showed significant increased risk of more than 30% to having dystocia at term irrespective if exposed solely to domestic violence or to history of violence. Kjaergaard et al.[10] has already presented overweight as a riskfactor for labour dystocia from the DDS [8-10].

Some potential obstetrical risk factors for dystocia were also analysed in relation to violence. Our findings showed that delivering a baby with a birth weight ≥ 3500 g was associated with dystocia at term without any association with exposure of violence. Yet, Kjærgaard et al.[8] have already shown on the DDS material that expecting a child with a birth weight > 4000 g was associated with increased risk of dystocia. Indeed, high birth weight as a predisposing factor for prolonged labour and labour dystocia is well-described in the literature [31,32]. Women exposed to violence more often give birth to low birth weight babies [20,22,24]. However, birth weight is probably not the sole explanation for labour dystocia, and women may have prolonged second stage without any correlation to birth weight [45]. It should also be noted that some studies have found no association between violence and low birth weight [14,46]. Furthermore, unknown factors such as psychosocial stress may also have some importance in this context. However, Nystedt et al. [47] could not find a link between a low level of psychosocial resources in early pregnancy and increased risk for prolonged labour. The etiology of the diagnosis labour dystocia appears to be multifaceted and therefore complex.

In addition, although instrumental delivery is a well-known independent consequence of dystocia, [4,6] we did not find any association between instrumental delivery and experience of violence with labour dystocia. Women with labour dystocia had significantly increased risk for instrumental deliveries, irrespective of exposure to violence or not, a finding which is unremarkable. Previous studies have found that women reporting physical violence during pregnancy are more likely to be delivered by caesarean section than those who are not exposed to physical violence [25,48]. However, it is important to keep in mind that in the current sample, only nulliparous women at term were included and thus all premature deliveries were excluded.

### Methodological discussion

The results of this study might potentially be biased due to selection or misclassification. However, we do not find any reason to believe that systematic selection bias or misclassification occurred. The current cohort design based upon prospectively collected data enabled the comparison of risk of labour dystocia among women exposed and un-exposed to violence during the same time period. The population in this study consisted only of nulliparous women which made the cohort a homogeneous group in that respect. Also, the concept 'dystocia' was very well defined, in accordance with ACOG criteria for dystocia in labour's second stage [6] and with the criteria for dystocia in the first and second stage described by the Danish Society for Obstetrics and Gynecology, [39,40] which means that the composition of the group defined with labour dystocia is homogeneous. However, our results raise the question as to whether these criteria for labour dystocia are relevant for the diagnosis. Labour dystocia is still a poorly defined phenomenon which might be categorized with respect to clinical diagnosis [12]. It may well be that the current definition with a time span of four hours is too short, and therefore the prevalence of dystocia may be overestimated. The use of a lengthier time criteria might lead to a reduced number of cases diagnosed as dystocia, but would probably yield a more accurate estimate. The extent to which this in turn might lead to a stronger association between experienced violence and labour dystocia is unknown.

The internal non-response rate of the questions about violence was only 0.5% that is, only 14 women in this cohort did not answer the violence questions at all. The limited number of women with missing information on violence exposure is unlikely to have affected the results in any major way, and we can only speculate as to whether these women were exposed to violence or not. However, as mentioned above, technical errors due to the use of the internet for data collection (40% of the answers at baseline) provided only two alternatives for answers regarding violence exposure, i.e. 'yes earlier', or 'no never', instead of three alternatives. Misclassification of responses could potentially have led to an underreporting of exposure to violence during pregnancy at term. MacMillan et al.[49] found that computer-based screening did not increase prevalence, and that written screening methods yielded fewest missing data.

The questions measuring violence used for this sub-study have been previously validated and used in a Danish general population [42]. However, since the questions have not been adapted to a pregnant cohort before, this may have influenced the findings obtained. Further, it is possible that the rather broad time frame for experienced violence investigated in the current study is not relevant for a study of obstetric outcome. However, according to Eberhard-Gran et al., [37] history of sexual violence in adult life is associated with an increased risk of extreme fear during labour. In our hypothetical model excessive stress, fear and anxiety are related to dysfunctional labour. Screening for violence is not a routine in all countries. If it could be known for the midwife and the obstetrician prior to delivery that the woman had been exposed to excessive stress due to domestic violence before or during pregnancy, then health care practitioners could provide closer monitoring throughout pregnancy and during delivery. The caring process could be more carefully scrutinised to the unique woman's needs. However, the extent to which closer monitoring would decrease risk for labour dystocia is still an unanswered question.

## Conclusions

The hypothesis that nulliparous women who have been exposed to violence are more prone to labour dystocia during childbirth at term has not been confirmed. Due to the current scarcity of studies exploring a possible association between violence and labour dystocia, two major contributors to adverse maternal and fetal outcome, the extent to which a relationship might exist would need further investigation. In this regard, it would also be beneficial if the criteria for the definition dystocia could be further evaluated.

## Competing interests

The authors declare that they have no competing interests.

## Authors' contributions

All authors contributed to the planning of the study. Analyses were planned by all authors. HF performed the analysis and all authors interpreted the results. HF wrote the drafts of the manuscript, which the other authors commented on and discussed. All authors approved the final manuscript.

## Pre-publication history

The pre-publication history for this paper can be accessed here:

http://www.biomedcentral.com/1471-2393/11/14/prepub

## Supplementary Material

Additional file 1 **Appendix**. Questions concerning violence used in the current study. Click here for file
