# Supplementary material for: A multi-centre cohort study shows no association between experienced violence and labour dystocia in nulliparous women at term
Source: BMC Pregnancy Childbirth. 2011 Feb 21;11:14. doi: 10.1186/1471-2393-11-14 (PMC3052209; doi:10.1186/1471-2393-11-14)
Supplement: Additional file 1 — Appendix. Questions concerning violence used in the current study. [file 1471-2393-11-14-S1.DOC]

Additional file 1. Questions concerning violence used in the current study. [8-10]

| 1. Have you ever been exposed to threat of violence? | Yes during this yes earlier no, never  pregnancy  □ □ □ |
| --- | --- |
| 2. Have you ever been pushed, shaken or struck lightly? | □ □ □ |
| 3. Have you ever been kicked, struck with a fist or object? | □ □ □ |
| 4. Have you ever been thrown against furniture, into walls, down stairs or similar? | □ □ □ |
| 5. Have you ever been strangulated, attempted assault with a knife or firearm? | □ □ □ |
| 6. Have you ever been exposed to another form of violence? | □ □ □ |
| 7. Have you ever been exposed to threat of sexual violence? | □ □ □ |
| 8. Have you ever been exposed to accomplished sexual violence? | □ □ □ |
| If you have answered yes to one or more of above questions about violence and sexual violence | |
| 9. By, whom was the violence perpetrated? |  |
| Your husband/Co-habitant | □ |
| A person you knew very well from your family | □ |
| A person you knew very well (not family member) | □ |
| A person you knew superficially (family or other) | □ |
| A person you did not know | □ |
| If there is something you really want to add to the questions about violence and assault you can write it down here.  ___________________________________________________________________________________  ___________________________________________________________________________________  ___________________________________________________________________________________  ___________________________________________________________________________________  ___________________________________________________________________________________ | |
